# Leucine-rich repeat kinase 2 and alpha-synuclein: intersecting pathways in the pathogenesis of Parkinson's disease?

**DOI:** 10.1186/1750-1326-6-6

**Published:** 2011-01-18

**Authors:** Elisa Greggio, Marco Bisaglia, Laura Civiero, Luigi Bubacco

**Affiliations:** 1 Department of Biology, University of Padova, Via U. Bassi 58/b, 35121, Padova, Italy

## Abstract

Although Parkinson's disease (PD) is generally a sporadic neurological disorder, the discovery of monogenic, hereditable forms of the disease has been crucial in delineating the molecular pathways that lead to this pathology. Genes responsible for familial PD can be ascribed to two categories based both on their mode of inheritance and their suggested biological function. Mutations in *parkin*, *PINK1 *and *DJ-1 *cause of recessive Parkinsonism, with a variable pathology often lacking the characteristic Lewy bodies (LBs) in the surviving neurons. Intriguingly, recent findings highlight a converging role of all these genes in mitochondria function, suggesting a common molecular pathway for recessive Parkinsonism. Mutations in a second group of genes, encoding alpha-synuclein (α-syn) and LRRK2, are transmitted in a dominant fashion and generally lead to LB pathology, with α-syn being the major component of these proteinaceous aggregates. In experimental systems, overexpression of mutant proteins is toxic, as predicted for dominant mutations, but the normal function of both proteins is still elusive. The fact that α-syn is heavily phosphorylated in LBs and that LRRK2 is a protein kinase, suggests that a link, not necessarily direct, exists between the two. What are the experimental data supporting a common molecular pathway for dominant PD genes? Do α-syn and LRRK2 target common molecules? Does LRRK2 act upstream of α-syn? In this review we will try to address these of questions based on the recent findings available in the literature.

## Introduction

Parkinson's disease (PD) is a common neurodegenerative disease historically classified as a sporadic disorder. The clinical phenotype of PD consists of motor dysfunctions including resting tremors, postural instability and bradykinesia. Non-motor manifestations such as autonomic and cognitive dysfunction are also recognized as part of the syndrome. Two histopathological features define the disease. First, there is a progressive degeneration of dopaminergic projections from the *substantia nigra pars compacta *(SNpc) to the striatum. These neurons are pigmented as they contain cytoplasmic neuromelanin, which accumulates in an age-dependent manner. When the first motor symptoms appear, the SNpc is already severely depigmented with over 70% of dopamine-producing cells lost. A second neuropathological event is the deposition of proteinaceous inclusions termed Lewy bodies (LBs) in the surviving neurons. LBs are predominantly made up of the small presynaptic protein α-syn [1], which is used as a marker for the progression of the disease.

The discovery of monogenic forms of PD marked a revolution in our understanding of the molecular mechanisms underlying this pathology. The big advantage of studying a genetic disorder compared to a sporadic syndrome is that we can use engineered cellular and animal models carrying the mutant gene to define pathological pathways. In 1997, mutations in the gene *SNCA*, encoding α-syn, were identified as cause of dominantly inherited PD [2]. Beta-sheet-rich fibrillar forms of α-syn aggregates represent the main constituents of LBs in PD and several other LB diseases [1]. Recently, polymorphisms around *SNCA *have been associated with increased risk of sporadic PD, indicating that the gene is also an important risk factor for the disease [3,4]. After *SNCA*, a number of additional genes have been linked to PD. Mutations in three genes, coding for parkin, DJ-1 and PINK1, are the cause of recessive forms of parkinsonism [5-7]. Interestingly, the major common functional effects of all three genes relate to mitochondrial function and oxidative damage, suggesting a common pathway for recessive parkinsonism. In 2004, mutations in the gene coding for the protein Leucine-rich repeat kinase 2 (LRRK2) were shown to cause an autosomal dominant form of PD [8,9] with a clinical presentation and disease onset very similar to the sporadic disorder. *Leucine-rich repeat kinase 2 *mutations account for 1-40% of total PD cases depending on the population under study [10], suggesting that they are also a risk factor for the disease.

Because α-syn and LRRK2 are implicated in both genetic and sporadic PD, understanding the physiological and pathological functions of these proteins may provide an excellent opportunity to gain insights into the sporadic disease, with obvious therapeutic implications.

In this review we will discuss some of the recent literature on the two genes that are known to cause dominantly inherited PD, namely *Leucine-rich repeat kinase 2 *and *SNCA*, focusing on the possible intersecting pathways between these two players.

## α-Syn: physiological and pathological role

**Figure 1:**
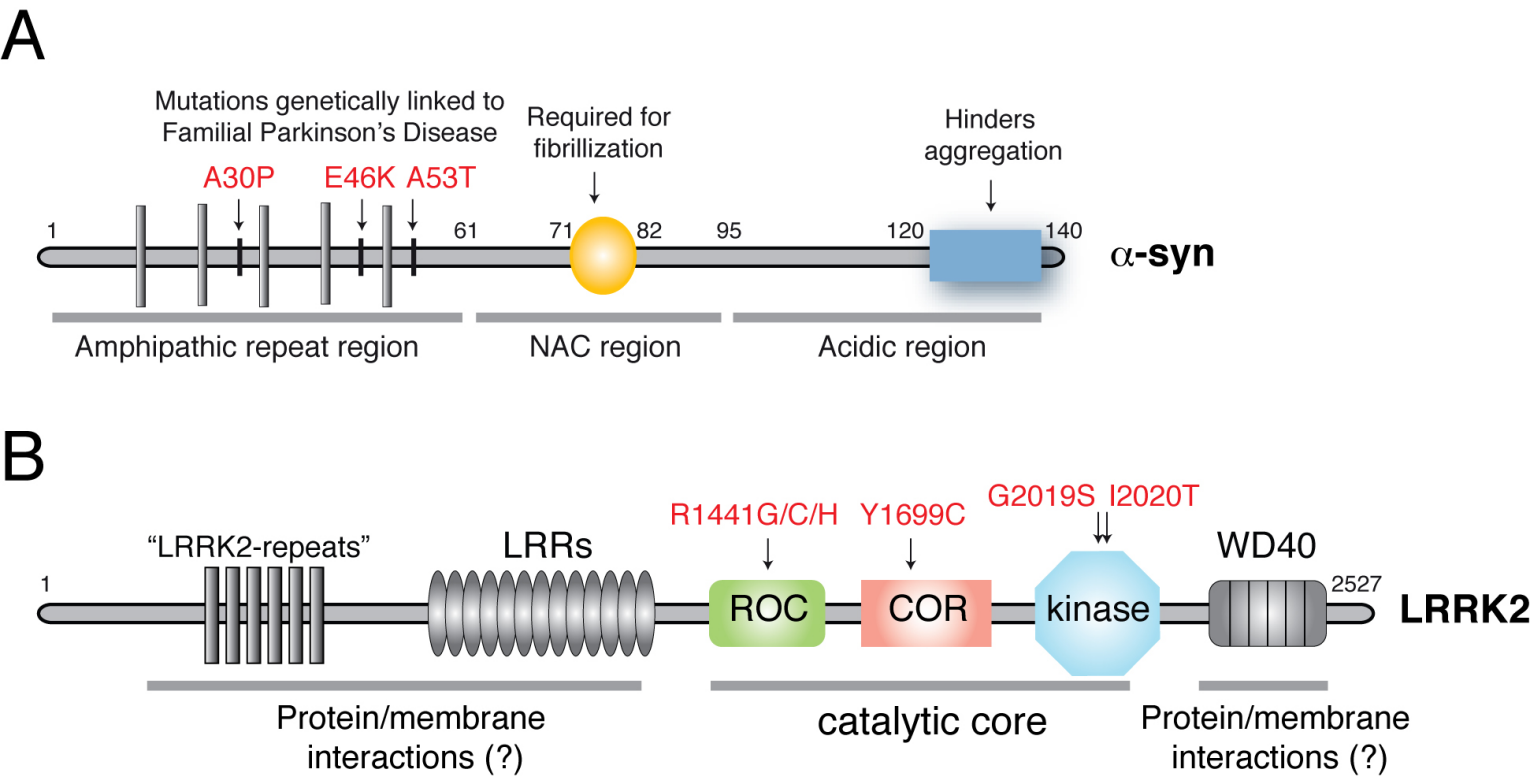
**(A) Schematic of α-syn domains (NAC, non-amyloid β component)**. (B) Schematic of LRRK2 domains (LRRs, Leucine-rich repeats; ROC, Ras Of Complex proteins; COR, C-terminus Of ROC).

The discovery of mutations in *SNCA *were the first evidence of a genetic cause for PD [2]. Three point mutations [2,11,12] as well as gene triplication [12] and duplication [13] have been linked to a form of parkinsonism similar to the sporadic syndrome. α-Syn is a 140-amino acid protein enriched in the presynaptic terminals of neurons in the central nervous system [14], where it has been associated with a specific subpopulation of synaptic vesicles [15] and with the lipid rafts of the plasma membrane [16]. The N-terminal region of the protein contains a number of imperfect repeats, with the consensus motif KTKEGV, which strongly resembles that found in the amphipathic helices of the exchangeable apolipoproteins [17]. The central hydrophobic region of α-syn, called NAC (non-amyloid component), has been suggested to be responsible for the aggregation process [18] while the acidic C-terminal region has been shown to regulate fibril formation [19] (figure 1A). Several studies suggest that α-syn exists in equilibrium between a cytosolic unfolded conformation and a membrane-bound, alpha-helical structure [20,21]. Interaction of α-syn with membranes has been extensively characterized *in vitro *using micelles and small unilamellar vescicles made of different phospholipids (see [22] for a review).

Although its precise function is still elusive, its subcellular localization within the nerve terminal and its ability to interact with membranes, suggest that α-syn may play a role in regulating vesicle dynamics and trafficking at the presynaptic terminal and in brain lipid metabolism [23,24]. A recent study by Burrè and co-workers nicely demonstrated that α-syn regulates the release of neurotrasmitters at the pre-synaptic terminal by promoting the assembly of the SNARE complex [25]. In addition, α-syn seems to modulate intracellular DA concentration through interactions with proteins that regulate DA synthesis and uptake, such as tyrosine hydroxylase [26], the aromatic amino acid decarboxylase [27] and plasma membrane dopamine transporter [28]. Interestingly, α-syn knock-out mice lack an obvious phenotype, suggesting that the protein does not play a crucial role in the development or neuronal maintenance [29-31]. Only a triple transgenic mouse lacking α-, β-, and γ-syn shows alterations in synaptic structure and transmission, age-dependent neuronal dysfunction and diminished survival [32]. These observations indicate that α-syn-induced neurodegeneration may not be due to a loss of function of the protein. In contrast to the minimal phenotype of α-syn knockout mice, recent works have shown that the overexpression of α-syn produces considerable toxicity by affecting synaptic transmission. The available evidence strongly suggests that inhibition of neurotransmitter release is the overall pathologic mechanism induced by excessive α-syn [33-35]. Then how does mutant α-syn cause PD? A dose-dependent toxicity of α-syn seen in duplication/triplication cases with an additional effect of homozygosity [36] plus the presence of the protein in LBs support the idea that the pathological mechanism of mutant α-syn is through a gain of function. In support of this notion, it has been widely demonstrated that α-syn is prone to aggregate into amyloid-like, beta-sheet fibrils [22] and fibrillisation is augmented in the presence of mutations or elevated protein levels [37-39].

Increasing evidence also suggests that phosphorylation may play an important role in modulating α-syn aggregation, LB formation, and toxicity [40]. It has been shown that α-syn deposited in LBs is highly phosphorylated at serine 129 [41,42] and serine 87 [43]. However, it is still unclear whether phosphorylation enhances or protects against α-syn toxicity *in vivo*. The role of phosphorylation in promoting or inhibiting fibril formation remains controversial. Phosphorylation at serine 129 has been reported to promote fibril formation more readily than unmodified protein, *in vitro *[41], but inhibition of oligomerization and fibril formation has been also described for serine 87 or serine 129 phosphorylated α-syn. An additional study utilizing an *in vivo *model suggests a lack of correlation between phosphorylation at Ser-129 and the level of α-syn fibrillation [44]. Clearly, more research is needed for a coherent view of how phosphorylation alters the physiochemical properties of α-syn to emerge.

One interesting property of α-syn is its ability to propagate from cell to cell. It has been shown that a proportion of α-syn and its aggregates are secreted from neuronal cells via exocytosis [45]. In addition, two studies showed host-to-graft propagation of α-syn-positive LB pathology in long-term embryonic nigral transplants in PD [46,47]. *In vitro*, cultured cells and neurons are capable of taking up α-syn aggregates via endocytosis [48] and this observation led to the development of cellular models of α-syn aggregation [49,50]. It is thought that exogenous fibrillar α-syn seeds the formation of LB-like inclusions by incorporating soluble monomeric proteins, in a process possibly analogous to the infectious propagation of prions [49]. Taken together, all these observations strongly support the notion that the presence of fibrils of α-syn represents a noxious event that leads to the pathological consequences observed in PD.

## LRRK2: a signaling protein that is toxic when mutated

In 2002 Funayama and collaborators reported a new genetic linkage to dominant inherited PD [51]. Two years later, independent groups not only described additional families with linkage at the same chromosomal locus but also found that the gene responsible for this familial form of PD was *Leucine-rich repeat kinase 2 *[8,9]. In particular one mutation, the glycine to serine substitution at position 2019, was soon recognized to be a very common cause of PD being present in 1 to 40% (depending on the population) of all PD cases, familial and sporadic. Pathological information of LRRK2 cases is available and suggests a quite variable pathology ranging from typical sporadic pathology with LBs, to ubiquitin, tau and/or TDP-43-positive inclusions, to pure nigral degeneration (reviewed in [52]). Although the majority of cases present with pathological and clinical features undistinguishable from idiopathic PD, a recognized variability in different LRRK2 mutation carriers may suggest that LRRK2 acts upstream of α-syn and other proteins implicated in the neurodegeneration associated with protein deposition. Therefore, understanding LRRK2 function might illuminate the pathological pathways that lead to α-syn deposition and on mechanisms at work in other LB disorders.

While the pathogenic impact of mutant α-syn is, at least in part, understood, the mechanism by which mutant LRRK2 causes PD is less clear. LRRK2 is a large, multidomain GTPase/kinase protein (figure 1B) that undergoes autophosphorylation *in vitro *[53-56]. Interestingly, pathological mutations cluster within the two enzymatic domains [57], suggesting the possibility that altered signaling is implicated in the disease. Kinase activity is required for mutant proteins to be toxic, at least in neuronal cell models [55,58], further supporting the notion that alteration of LRRK2 signaling might have pathological implications. Clues should come from the effects of pathological mutations on protein activity. Only one mutation, the G2019S located in the activation loop of the kinase domain, clearly increases kinase activity (reviewed in [57]), while other mutations do not convincingly do so [57]. Since blocking kinase function rescues the toxicity observed in primary neurons, it is not clear why toxicity is prevented for mutants with unaltered kinase activity. One possible interpretation is that the kinase domain regulates the GTPase/ROC (Ras Of Complex proteins) domain and ROC is the signal output of LRRK2 involved in the pathogenic process. In support of this hypothesis, we and others have shown that the kinase phosphorylates its own ROC domain [59-61], opening the possibility of an intramolecular mechanism of regulation. As mutations in the ROC/GTPase domain decrease the ability of LRRK2 to hydrolyze GTP [62,63], it can be speculated that the G2019S mutation indirectly affects the catalytic properties of ROC through increased phosphorylation. However, the only experimental measurement of the GTPase activity for the G2019S mutant suggests that there is no significant change compared to the wild-type [64], indicating that different mutations might act in different ways and ultimately converge to a common pathological phenotype. This is also supported by the different pathologies observed among different mutants. Interestingly, pathological mutations associated with PD have not been described in the paralogous protein LRRK1 [65] and analogous LRRK2 mutations are innocuous when artificially introduced in LRRK1 [65].

What is LRRK2's physiological function? LRRK2 has been suggested to play a role in the control and maintenance of neurite length [66-69], in vesicle endocytosis through interaction with Rab5a [70] and vesicle sorting between axons and dendrites [71], in activation of apoptosis through interaction with death adaptor Fas-associated protein with death domain (FADD) [72], and in controlling protein translation through phosphorylation of 4E-BP1 [73] and interaction with the microRNA pathway to regulate protein synthesis [74]. Several groups also reported that LRRK2 interacts with alpha and beta tubulins, the microtubule's building blocks [75-77], suggesting that LRRK2 may play a role in cytoskeleton dynamics. Interestingly, LRRK2 localization with microtubules is enhanced in the presence of the potent LRRK2 inhibitor H-1152 [78], indicating that this interaction is dependent on kinase activity.

What do we know about the mechanism of LRRK2 mediated neurodegeneration? The observation that homozygous cases with G2019S mutation have a clinical presentation undistinguishable from the heterozygotes [79] suggests that the mechanism of LRRK2 pathogenicity may not be a consequence of increased protein activity. This is in apparent contrast with the observation that mutations cause differential effects on protein activity, as discussed above. It is possible that the amount of altered activity is no longer important above a certain threshold (i.e. downstream targets are limiting factors) and therefore having 50% or 100% of mutant molecules does not make any difference in terms of cellular effect. This important but still unresolved issue will become more clear when robust physiological substrates of LRRK2 are identified. Although a few LRRK2 substrates have been described [73,77,80-83], we are still awaiting reproducible and physiologically relevant substrates.

Could mutant LRRK2 act with a dominant negative mechanism? A requirement for a mutant protein to operate as dominant negative is that the protein exerts its biological function within a homo or hetero-complex. Several independent groups have now demonstrated that LRRK2 is predominantly a dimer under native conditions [56,84-87]. Interestingly, a recent paper by Tong and collaborators [88] shows that loss of LRRK2 causes accumulation of α-syn, increased autophagy and cell death in kidneys of aged mice. These data may indicate that mutations cause loss of function through a dominant negative mechanism. However, why the effect was seen specifically in the kidneys and not in the brain, which is the relevant tissue for the neurodegenerative process, needs to be further elucidated. One explanation suggested by the authors is that the renal tissue has almost undetectable levels of LRRK1 mRNA expression and therefore LRRK1 may not compensate loss of LRRK2 function. What happens when both LRRK1 and LRRK2 are lost? Double-knockout mice for LRRKs have not been reported in the literature and one possibility is that these mice are not viable. In another study, Sheng et al. [89] observed that LRRK2 knock-out in zebrafish by morpholinos is embryonically lethal while deletion of the C-terminal WD40 domain induces a parkinsonism-like loss of neurons and locomotive defects, indicating that LRRK2 function is essential for neuronal survival. This observation supports the notion that LRRK2 mutations cause a loss of protein function.

Interestingly, a few studies reported a number of PD cases with LRRK2 positive staining in LBs of *post-mortem *sections using different antibodies against LRRK2 [55,90-94]. However, some inconsistency between different studies [95,96] makes the data difficult to interpret, although the lack of reliable immunological tools to detect LRRK2 within cells or tissues is currently a major limitation in the field. Furthermore, different groups reported a mutant-specific tendency of LRRK2 to accumulate into inclusion bodies when overexpressed in cell lines and primary neuronal cultures [55,66,96,97]. Sequestration of mutant proteins into inclusion bodies could lead to a loss or a gain of function depending if these aggregates are toxic for the cells. Although the main criticism to this observation is that protein overexpression could be a misleading approach as non-physiological levels of a given protein my cause artefactual aggregation, the clear-cut phenotype observed between wild-type and mutant LRRK2 highlights the importance of further investigating LRRK2 aggregation properties and products. Waxman and collaborators observed that LRRK2 inclusions do not recruit α-syn when both proteins are co-overexpressed in cell systems [96], hinting that LRRK2 and α-syn deposition might be two independent processes.

Although LRRK2 pathological function is still unclear, a unifying theme of altered dopaminergic neurotransmission is emerging, based on two transgenic mouse models carrying different pathological LRRK2 mutations, suggesting that LRRK2 normal function is crucial at the synaptic level, as proposed by Lee *et al*., [98].

## LRRK2 and α-syn: intersecting pathways?

As discussed above, understanding LRRK2 and α-syn pathways may shed light on the mechanisms that underlie sporadic PD. Is there any evidence that suggests a functional link between α-syn and LRRK2? α-Syn deposited in LBs is highly phosphorylated at serine 129 [41] and phosphorylated proteins seem to be more prone to aggregation *in vitro *[41], suggesting that abnormally high levels of phosphorylated proteins may trigger the neurodegenerative process. A simple scenario is that LRRK2 is the kinase that mediates phosphorylation of α-syn. However, only one report showed that recombinant α-syn is directly phosphorylated by cell lysates overexpressing LRRK2 from HEK293 cells [99], while there is no evidence that LRRK2 causes increased α-syn phosphorylation in cell or animal systems. It would be of particular interest to investigate whether pathological brain tissue from LRRK2 cases display increased levels of phosphorylated α-syn. One study reported that LRRK2 induces α-syn expression *via *the extracellular signal-regulated kinase pathway, although the effect is modest [100]. Qing and collaborators [101] successfully co-immunoprecipitated LRRK2 and α-syn from pathological tissue of diffuse LB cases or from HEK293 cells when exposed to oxidative stress. These data are quite interesting as they hint the possibility that the two proteins localize upon stress to the same cellular compartment where they participate in a common biological process and LRRK2 kinase activity might, directly or indirectly, influence α-syn phosphorylation state.

Another possibility is that LRRK2 accelerates the toxicity of α-syn *via *a different mechanism other than phosphorylation. For instance, the role of the GTPase activity of LRRK2 is still a relatively unexplored field and, to date, no LRRK2 partners specific for the GTP-bound state of the protein have been reported. Important clues on the α-syn/LRRK2 interplay come from a recent work by Cai and collaborators [102]. Using the tetracycline-controlled transactivator system, they generated a number of inducible transgenic mice overexpressing the human A53T mutant form of α-syn, which they crossed with mice transgenic for human LRRK2, including wild-type, G2019S, or kinase domain-deletion mutant in the adult forebrain. They found that co-expression of LRRK2 with A53T α-syn dramatically accelerates the neurodegenerative process in a dose dependent manner and independently from the LRRK2 genotype, suggesting the kinase activity is not important for the observed phenotypes. LRRK2 expression led to impairment of microtubule dynamic, Golgi and mitochondria defects. Strikingly, they observed an age-dependent accumulation of α-syn in double transgenic mice, suggesting that LRRK2 acts upstream of α-syn depositions. Interestingly, loss of LRRK2 (by transgenic knockout) alleviates these phenotypes. Although these data are quite illuminating in establishing a functional link between LRRK2 and α-syn, whether this also happens in the DA neurons of the midbrain, is an important question that needs to be addressed.

Are LRRK2 and α-syn physically found in the same compartments? We have already discussed that α-syn interacts with membranes where it acquires an alpha-helical conformation. LRRK2 is also found in association with membranous structures [82,87,103,104]. It is associated with small vesicles and it interacts with Rab5 playing a possible role in vesicle endocytosis at the synaptic terminal [70]. This is an interesting aspect as it links LRRK2 and α-syn, which is also thought to play a role in synaptic vesicle recycling, as discussed above. Both LRRK2 and α-syn have also been proposed to interact with lipid rafts [16,105]. Lipid rafts are organized membrane microdomains that serve as platforms for intracellular signaling. These domains are organized by specialized scaffold proteins and one hypothesis is that LRRK2 could function as a scaffolding molecule mediating heterologous interactions through its predicted protein-protein interaction domains (i.e. Leucine-rich repeats and WD40 domain). Mutations could impair LRRK2 ability to properly organize the lipid raft with direct consequence on α-syn interactions with these domains.

In another study, Sakaguchi-Nakashima et al. [71] investigated the function of LRK-1, the *C. elegans *homolog of LRRK1/2. They proposed that LRK-1 controls a polarized sorting of synaptic vesicle proteins to the axons, excluding them from the dendrite-specific transport. It can be speculated that an altered axonal-transport due to the presence of pathological LRRK2 mutations, could lead to an over-accumulation of toxic proteins, like α-syn, in dopaminergic neurons.

**Figure 2:**
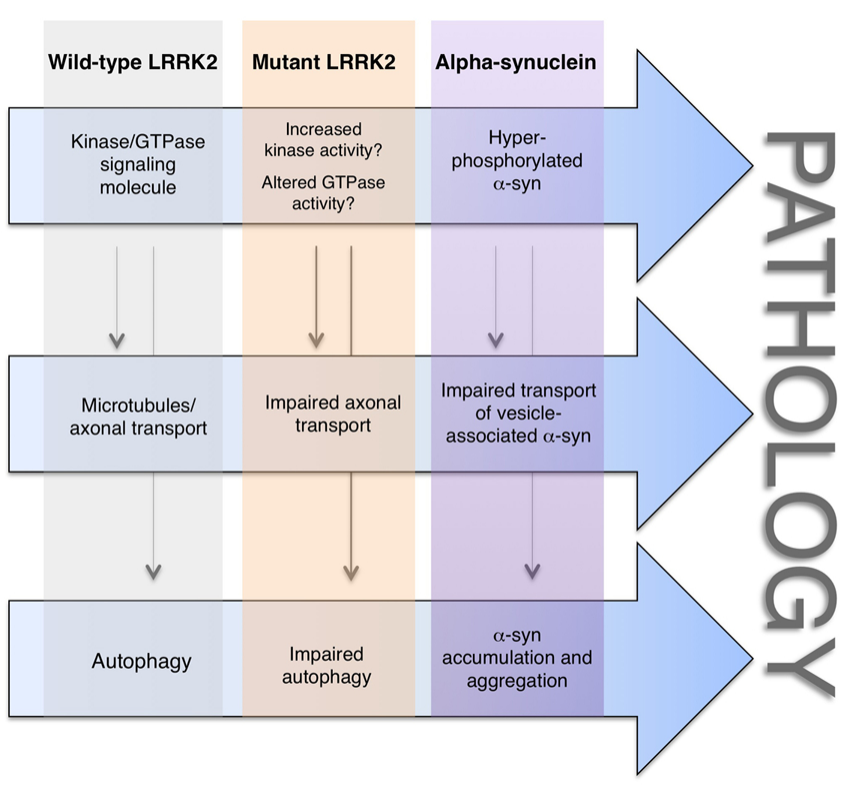
**Possible pathological pathways connecting LRRK2 and α-syn**.

Another set of observations linking LRRK2 and α-syn converge to microtubule dynamics and axonal transport. In the process of organelle transport along the axons, for example synaptic vesicles, binding of kinesin to microtubules requires the presence of JIPs (JNK interacting proteins) - a family of scaffolding molecules that adapt the binding of cargoes to kinesin [106]. Interestingly, JIPs have been proposed to interact with LRRK2 [107]. Furthermore, as JIPs bind to the protein kinase MKK7, also LRRK2 is capable to interact with MKK7 [82]. By binding to tubulins, LRRK2 stabilizes microtubules *in vitro *[77]. A possible readout is that LRRK2 is upstream of α-syn and acts as a scaffolding signaling molecule required for organelle transport and microtubule assembly and stability. On the other hand, several experimental evidences indicate that LRRK2 causes tau hyperphosphorylation, an event that is believed to induce destabilization of microtubules [66,108-110]. Altered LRRK2 function by mutations may lead to increased microtubule destabilization and, as a result, improper transport of vesicle bound-α-syn with consequent protein accumulation and, in turn, cell death (Figure 2).

What is LRRK2 role in signal transduction? There is mounting evidence that LRRK2 might be involved in a mitogen-activated protein kinase (MAPK)-related pathway. Overexpression of LRRK2 increases ERK1/2 phosphorylation [100,111] and LRRK2 interacts with and phosphorylates *in vitro *the MAPKKs MKK3, 6 and 7 [81,82]. Also, LRRK2 is involved in the regulation of neurite outgrowth and mutant LRRK2 causes neurite shrinkage [66,67]. Since MAPK pathway may play a role in neurite outgrowth [112], it is possible that LRRK2 modulates neurite morphology and length through MEK/ERK phosphorylation. Interestingly, a recent study uncovered that LRRK1, the close LRRK2 homologue, is part of a complex including Grb2/Gab2/Shc1 (adaptor proteins for Ras activation) and that this signal integrator complex is involved in the balance of cellular stress responses (influencing both ERK and JNK) only if LRRK1 is functional [113]. Based on these observations, one could speculate that unbalanced phosphorylation of downstream components of a MAPK-related pathway, as a consequence LRRK2 mutations, could in turn affect the phosphorylation state of α-syn.

An interesting work by Sancho and collaborators [114] places LRRK2 in the *Wnt *(Wingless/Int) signaling pathway by a direct interaction with Dishevelled (DVL) proteins through the ROC-COR domain. Interestingly, LRRK2 enhances tau phosphorylation through the downstream target of DVLs GSK-3beta [110] while (i) tau phosphorylation by GSK3-beta is α-syn-dependent [115] and (ii) the phosphorylation of the α-syn interacting protein synphilin-1 is mediated by GSK3-beta [116]. Furthermore, Li and co-workers observed increased phosphorylation of Tau in a mouse model of mutant LRRK2 [108]. Taken together, these observations further expand an emerging link between PD and tau, as variations in the *MAPT *gene are associated with increased risk of PD [3] and LRRK2 and tau, which, as discussed earlier, is deposited in a number of LRRK2 cases.

Another set of interesting data links the chaperone proteins 14-3-3 with both LRRK2 and α-syn. 14-3-3 proteins, a highly conserved chaperone family consisting of seven known mammalian isoforms, regulate a variety of cellular processes including intracellular trafficking and protein interaction [117]. They have been shown to form a complex with α-syn [118] and to co-localize with LBs in PD and diffuse LB disease [119]. Furthermore, 14-3-3 proteins protect against neurotoxicity and aggregation of α-syn in a number of cellular and animal models [120]. Two recent papers have reported that 14-3-3 also interacts with LRRK2 by binding two phosphorylated serine residues (S910 and S935) [78,97]. Interestingly, site-specific mutagenesis of the two serine residues or pharmacological inhibition of LRRK2 with H-1152, not only abolished the binding of LRRK2 with 14-3-3, but also caused the proteins to accumulate into aggresome-like structure in HEK293T cells. This aggregation phenotype was also observed in a number of pathological LRRK2 mutants (unable to bind to 14-3-3), strongly suggesting that 14-3-3 binding prevents LRRK2 aggregation. Collectively, these observations suggest that mutations in α-syn and LRRK2 or high dose of α-syn may disrupt or alter the ability of 14-3-3 to keep both proteins properly soluble with consequent increase in protein aggregation and, in turn, neurotoxicity.

Another potential mechanism by which mutant LRRK2 could promote α-syn aggregation is through impairment of autophagy. There are few studies highlighting a role of LRRK2 in the autophagic pathway. LRRK2 null mice display impaired autophagy function, accumulation of α-syn in the kidneys and consequent cell death [88], suggesting that LRRK2 function is implicated in the autophagic pathway and, if mutations cause loss of function, they may impair autophagy. In HEK293 cells, expression of R1441C mutant causes impairment of autophagy by accumulation of autophagic vacuoles containing incompletely degraded material and increased levels of p62 [121]. Furthermore, overexpression of G2019S mutant in the neuroblastoma line SH-SY5Y not only resulted in increased autophagic vacuoles but also caused neurite shrinkage [68], perhaps suggesting that the two events are related. Interestingly, wild-type α-syn is selectively translocated into lysosomes for degradation by the chaperone-mediated autophagy (CMA) pathway in isolated liver lysosomes. The pathogenic A53T and A30P mutants bound to the receptor for this pathway on the lysosomal membrane, but appeared to act as uptake blockers, inhibiting both their own degradation and that of other substrates [122]. Moreover, CMA inhibition leads to an accumulation of soluble high molecular weight and detergent-insoluble species of α-syn, suggesting that CMA dysfunction may play a role in the generation of such aberrant species in PD [123]. Autophagy impairment caused by mutant LRRK2 may result in accumulation of misfolded α-syn similarly to the effect of α-syn mutations (Figure 2).

## Conclusions

The future key steps in the field will be the acquisition of a clearer picture of LRRK2 signaling, including specific substrates, the GTPase activating proteins (GAPs) and the guanine-exchange factors (GEFs), and the kinases and phosphatases that finely tune its function. This will also allow to better understand how LRRK2 signaling influences α-syn function, with important therapeutic implications being LRRK2 a protein kinase and therefore an excellent pharmacological target.

## Competing interests

The authors declare that they have no competing interests.

## Authors' contributions

EG, MB, LC and LB conceived and wrote the manuscript. All authors read and approved the final draft.
